# Communication-related aspects drive senior patients’ satisfaction with the process of decision-making in cancer therapy

**DOI:** 10.1038/s41598-026-51157-y

**Published:** 2026-04-30

**Authors:** Andreas Heidenreich, Rabea Fuchshofen, Susanne Elsner, Frank Gieseler, Alexander Katalinic, Joachim Hübner

**Affiliations:** 1 Institute of Social Medicine and Epidemiology, University of Luebeck, Ratzeburger Allee 160, 23560 Luebeck, Germany; 2 Clinic for Hematology and Oncology, University Hospital Schleswig-Holstein (UKSH), Luebeck, Germany; 3 Agency for Clinical Cancer Data of Lower Saxony, Oldenburg, Germany

**Keywords:** Cancer, Health care, Oncology

## Abstract

Treatment decisions in cancer care can be life-critical and significantly affect self-determination concerns. Therefore, it is of great importance that the decision-making process is set up in the interest and satisfaction of the cancer patient. In a survey of 2,011 patients aged ≥ 50 years with breast cancer or colorectal cancer, 72.1% reported high satisfaction with the treatment decision-making process. Multivariate logistic regression identified several independent predictors of higher satisfaction: receiving comprehensive information about the illness and therapy options (adjusted odds ratio (aOR) 5.37, 95% CI 3.89–8.52), strong personal involvement in decision-making (aOR 2.93, 95% CI 2.15–3.98), perceived concordance among treating physicians (aOR 2.01, 95% CI 1.49–2.71), higher self-efficacy (aOR 1.63, 95% CI 1.31–2.04), and greater social support (aOR 1.36, 95% CI 1.10–1.67). Conversely, frailty was associated with lower satisfaction (aOR 0.66, 95% CI 0.48–0.90). These findings suggest that patient satisfaction with decision-making may be enhanced when physicians provide thorough information, actively involve patients in decisions, and coordinate care consistently, while frail patients and those with low self-efficacy or limited social networks may benefit from targeted support.

**Supplementary Information:**

The online version contains supplementary material available at 10.1038/s41598-026-51157-y.

## Introduction

Making therapy decisions about cancer treatment is a complex process. The journey from diagnosis to treatment can involve navigating a web of medical advice, personal values, and emotional considerations. The last decades saw a general shift towards patient perspectives and preferences as the guiding principle in healthcare^1^. The traditional paternalistic model of medical decision-making is characterized by the belief that the physician’s judgment should dictate all treatment decisions^2^. This is increasingly being supplanted in modern medicine by the “shared decision-making” approach (SDM), which is considered an essential component of patient-centered health care. SDM describes the collaborative process in which patients and healthcare providers jointly make health decisions, integrating external evidence and the physician’s individual expertise with patient preferences and values^3^.

Beyond the ethically justified demand for patient-centeredness, there is evidence that patient involvement in treatment decisions measurably improves healthcare quality. Higher levels of SDM and patient-centered communication are associated with significantly higher patient satisfaction scores^4^. Even a physician’s positive attitude toward shared decision-making positively affects overall patient satisfaction^5^. Higher patient satisfaction, in turn, is associated with better survival in pancreatic cancer^6^, colorectal cancer^7^, and breast cancer^8^ after controlling for cancer stage. One possible explanation is that adequately involved patients have greater trust in the treatment’s benefits and, therefore, are more compliant with therapy^9^.

Decision-making in cancer care places particular demands on those involved. The life-threatening character of most cancers and the seriousness of treatment-related side effects may cause patients to experience fear and unease. Being expected to take an active role in their health decisions can put additional pressure on patients^10,11^. Older patients in particular frequently face challenges in medical communication^12^, and the management of complex cancers in Germany has been described as lacking cohesive interdisciplinary coordination, leaving patients without sufficient support^13^. A satisfactory decision-making process cannot be taken for granted, especially in this demographic, and must be facilitated by medical professionals.

Prior research has examined several patient-level factors in relation to cancer treatment decisions. Patient-perceived informedness and information exchange with physicians have been shown to predict decision satisfaction in registry-based studies of breast, prostate, and colorectal cancer patients^14^. Patients’ perceived involvement in treatment decisions has been associated with better outcomes in breast cancer, including higher quality of life, with insufficient involvement linked to decisional role regret^15^. Perceived social support – both tangible and emotional/informational – has been linked to higher self-efficacy^16^, and self-efficacy itself has been identified as a predictor of decision-making participation in older breast cancer patients^17^. Depression has been associated with lower patient satisfaction in cancer care^18^. Finally, frailty and polypharmacy are well-characterized determinants of treatment outcomes in geriatric oncology^19,20^, but have rarely been examined as predictors of satisfaction with the decision-making process itself – a gap that is particularly relevant for older patients, who may encounter distinct cognitive, physical, and social limitations that can shape their participation in treatment decisions.

In the present study, we survey senior (defined here as aged 50 years and older, i.e., outside the early-onset age range) breast cancer and colorectal cancer patients in Germany regarding their satisfaction with the therapy decision-making process and a broad set of candidate predictors spanning patient health status, autonomy and support, and knowledge- and communication-related factors.

## Methods

### Patient recruitment

We queried participants aged 50 years and older with colorectal cancer (ICD-10 C18, C19, C20) or breast cancer (ICD-10 C50) on their experiences as patients with cancer. We sought insight into their therapy decision-making situations and surrounding factors. Participants were recruited in six cooperating cancer centers in Germany and through the cancer registries of the German federal states Hamburg and Schleswig-Holstein. Patients in cancer centers were recruited from December 2018 to July 2019 during their inpatient stays. They were given an initial questionnaire (not included in this analysis) and received a follow-up questionnaire six months later, assuming primary therapy was completed. Participants identified via cancer registries were invited by postal mail to join the study and were sent the questionnaire. These registry-based patients received their cancer diagnosis between January 2018 and December 2019. Written informed consent was obtained from all participants for the use of questionnaire data and access to UICC staging data, either from the responsible cancer center or the cancer registry. The study was approved by the University of Luebeck’s ethics committee (decision: October 5, 2017; file: 17–288).

### Outcome measure

We measured patients’ satisfaction with therapy decision-making using a single item: “All in all, how satisfied are you with how treatment decisions were made?” The response options were presented on a Likert scale from 1 (“very unsatisfied”) to 7 (“very satisfied”). We dichotomized the outcome measure, classifying patients who chose scores of 6 or 7 as ‘highly satisfied’ and those who chose scores of 1 through 5 as ‘less satisfied.’ The choice of cut-off and its analytical implications are discussed below.

### Independent variables

This study was part of a larger project on adherence to therapy guidelines in elderly cancer patients. Findings from this project’s other studies have been published elsewhere^21–25^. We used a questionnaire that assesses various physical factors and comorbidities, social participation and support, the experience of the treatment process, and knowledge about the cancer and its treatment. We used established and validated instruments to assess frailty (Tilburg Frailty Indicator, TFI^26^, self-efficacy beliefs (Short Scale for Measuring General Self-efficacy Beliefs, ASKU^27,28^, and social support (Questionnaire on Social Support, FSozU-14^29^. We ensured the questionnaire’s comprehensibility by conducting a pretest with a cohort representative of our study population. An overview of the instruments and items used in this analysis, along with their operationalization, is provided in Table 1. Patient age, sex, cancer staging, and therapy modalities were derived from medical records or cancer registries.

**Table 1:** Questionnaire items and operationalization.

Item	Operationalization
Outcome measure	
Satisfaction with therapy decision-making: “All in all, how satisfied are you with how treatment decisions were made?”	7-point Likert scale, “very unsatisfied” to “very satisfied” dichotomization reflects high satisfaction (options 6 and 7)
Patient characteristics and ailments	
Need of care	“Pflegegrad” 1–5 according to German social code § 15 SGB XI and “No” dichotomized to reflect the existence of “Pflegegrad”
Depression diagnosed before cancer diagnosis	Yes/No
Regular intake of four or more medications	Yes/No
Frailty	Tilburg Frailty Indicator, dichotomized per instrument instructions (≥ 5)
Patient autonomy and support	
Independently mobile by access and ability to use car or public transport	Yes/No
Self-efficacy expectations	ASKU self-efficacy scale, continuous
Social support	FSoZU social-support scale, continuous
Confidant available for advice on therapy decision	Yes/No
Knowledge- and communication-related factors	
Knowledge of guidelines: “Do you know medical guidelines or patient guidelines for treating cancer?”	Answer options: “don’t know them,” “know them but didn’t read them,” “read them”
Information about illness and therapy options “I was well-informed about my illness and treatment options.”	5-point Likert scale, “strongly disagree” to “strongly agree” dichotomization reflects agreement to statement (options 4 and 5)
Inclusion in treatment decision-making: “I was well-included in treatment decisions.”	5-point Likert scale, “strongly disagree” to “strongly agree” dichotomization reflects agreement to statement (options 4 and 5)
Perceived concordance of physicians’ actions: “My doctors work hand-in-hand” and “between the participating doctors, the flow of information works well”	Construct of two 5-point Likert scales, “strongly disagree” to “strongly agree” Cronbach’s alpha: 0.94 dichotomization at ≥ 4.0 reflects agreement

### Statistical analysis

We used descriptive statistics to explore our study sample. We conducted univariate analyses to calculate Odds Ratios (OR) to assess the relationship between each candidate variable and the outcome measure, with OR > 1 indicating higher satisfaction. We set a threshold of *P* < 0.1 in univariate regression to identify variables of interest for inclusion in multivariate logistic regression to estimate adjusted Odds Ratios (aOR).

Qualified variables were included as candidate predictors in an initial full model. To mitigate the risk of overfitting and identify the most parsimonious set of predictors, we employed a systematic, bidirectional selection process based on the Akaike Information Criterion (AIC)^30–32^, evaluating both the removal and addition of variables to retain those that significantly improved model fit while excluding those with minimal or redundant contributions. Multicollinearity in the models was assessed using Generalized Variance Inflation Factors (GVIF). We conducted sensitivity analyses stratified by sex to determine the stability of the predictors identified in our general model. For multivariate analysis, the significance level was set at α = 0.05 (5%).

Given the substantial sex imbalance in the sample toward women, we performed sex-stratified regression analyses to evaluate whether the predictors identified as significant in the full-sample analysis remained significant when analyzed separately for men and women.

All statistical analyses were performed using the R software and programming language, version 4.2.2^33^.

## Results

**Fig. 1:**
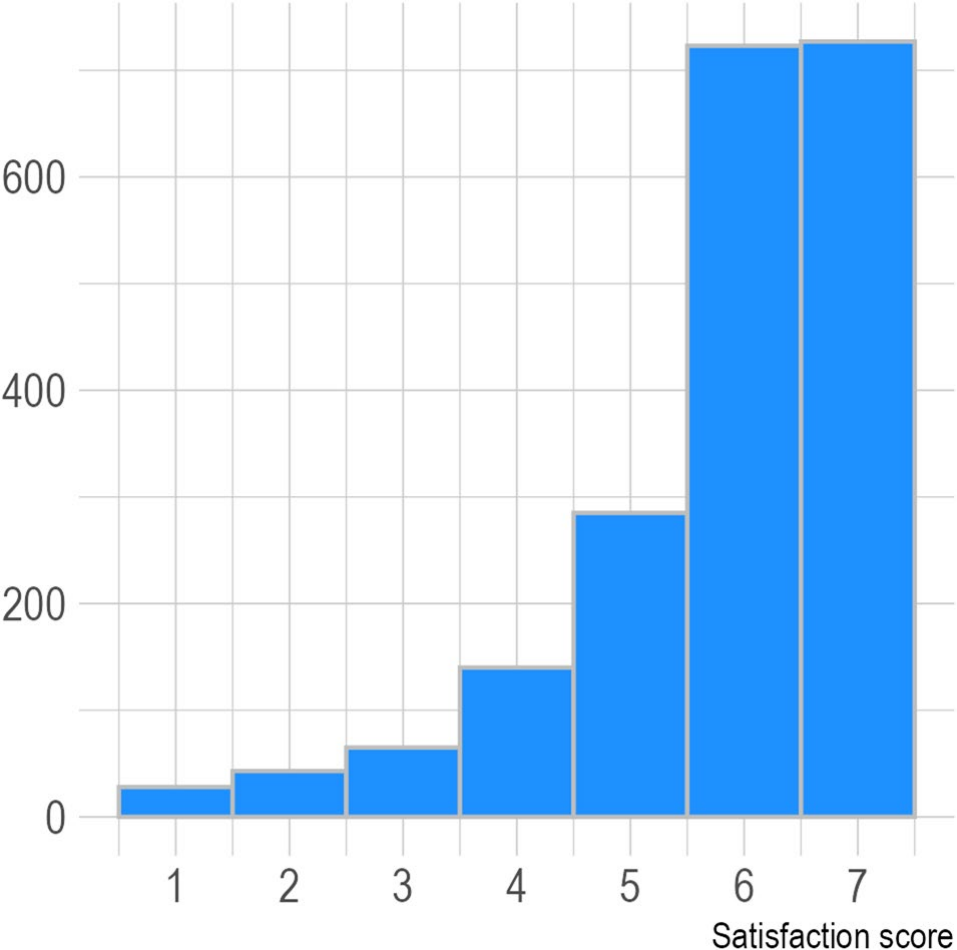
Distribution of the outcome variable, “Satisfaction with therapy decision-making,” with 1 representing lowest satisfaction, 7 representing highest satisfaction.

We surveyed 5,077 patients via cancer registries, of which 1,900 responded to the outcome measure. An additional 111 responses were obtained from patients recruited via cancer centers. Of these 2,011 participants, 1,450 (72.1%) chose one of the two highest scores on the 7-point satisfaction scale and were thus considered “highly satisfied.” The distribution of satisfaction scores is displayed in Fig. 1. To assess the robustness of findings under the chosen dichotomization, sensitivity analyses were conducted using alternative cut-offs (1–4 vs. 5–7; 1–6 vs. 7), an ordinal proportional-odds model, and a linear regression treating the outcome as continuous. Results were largely consistent across specifications with respect to the direction and significance of associations.

Respondents were predominantly women (80.7%) and diagnosed with breast cancer (67.0%). The median age at diagnosis was 69 years. Cancer was mostly in earlier stages, with 82.2% of patients being diagnosed at UICC stages I and II. Patient characteristics are displayed in Table 2.

**Table 2:** Patient characteristics.

	Overall, *N* = 2,011^*1*^	Less satisfied, *N* = 561^*1*^	Highly satisfied, *N* = 1,450^*1*^
Age	69 (60, 76)	68 (59, 77)	69 (60, 76)
Sex			
Female	1,619 (80.7%)	458 (82.4%)	1,161 (80.1%)
Male	386 (19.3%)	98 (17.6%)	288 (19.9%)
Cancer			
Breast cancer	1,348 (67.0%)	382 (68.1%)	966 (66.6%)
Colorectal cancer	663 (33.0%)	179 (31.9%)	484 (33.4%)
UICC staging			
Stage I	1,077 (57.1%)	290 (55.0%)	787 (58.0%)
Stage II	472 (25.0%)	124 (23.5%)	348 (25.6%)
Stage III	250 (13.3%)	84 (15.9%)	166 (12.2%)
Stage IV	86 (4.6%)	29 (5.5%)	57 (4.2%)
Frailty	786 (41.3%)	327 (60.8%)	459 (33.7%)
Medication	605 (30.3%)	218 (39.0%)	387 (26.9%)
Depression	325 (16.9%)	102 (19.4%)	223 (16.0%)
Need of care	152 (7.7%)	70 (12.8%)	82 (5.8%)

^1^ Median (IQR); n (%).

Univariate analysis of independent variables revealed that neither the patients’ age (OR: 1.00, 95% CI: 0.99 to 1.01), sex (OR: 1.16, 95% CI: 0.90 to 1.50), or tumor site (OR: 1.07, 95% CI: 0.87 to 1.32) was associated with their satisfaction regarding decision-making. Patients who deemed themselves well-informed about their illness and therapy options were more likely to be satisfied with the decision-making process (OR: 10.96, 95% CI: 8.31 to 14.57). Conversely, frailty was associated with lower satisfaction (OR: 0.33, 95% CI: 0.27 to 0.40). The results of the univariate regression analyses are displayed in Table 3.

**Table 3:** Univariate regression analysis.

	*N*	OR	95% CI	*P*
Patient characteristics and ailments				
Age	2011	1.00	0.99, 1.01	0.949
Sex	2005			0.249
Female		–	–	
Male		1.16	0.90, 1.50	
Cancer site	2011			0.528
Breast cancer		–	–	
Colorectal cancer		1.07	0.87, 1.32	
UICC staging	1885			**0.093**
Stage I		–	–	
Stage II		1.03	0.81, 1.32	
Stage III		0.73	0.54, 0.98	
Stage IV		0.72	0.46, 1.17	
Need of care	1972	0.41	0.30, 0.58	**< 0.001**
Depression diagnosed prior to cancer diagnosis	1920	0.79	0.61, 1.03	**0.084**
Regular intake of 4 + medications	1998	0.58	0.47, 0.71	**< 0.001**
Frailty (TFI)	1902	0.33	0.27, 0.40	**< 0.001**
Patient autonomy and support				
Independently mobile	1990	2.21	1.51, 3.23	**< 0.001**
Self-efficacy (ASKU scale)	1946	2.06	1.78, 2.39	**< 0.001**
Social support (FSozU scale)	1972	2.02	1.75, 2.33	**< 0.001**
Confidant available for support in consultations	1976	2.62	1.97, 3.48	**< 0.001**
Knowledge and communication-related factors				
Knowledge of treatment guidelines	1938			**< 0.001**
Not heard about it		–	–	
Heard about it		1.54	1.19, 1.99	
Read it		1.92	1.51, 2.43	
Informed about the illness and therapy options	1995	10.96	8.31, 14.57	**< 0.001**
Inclusion in therapy decision-making	1979	6.79	5.44, 8.48	**< 0.001**
Concordance of physicians’ actions	1928	4.12	3.32, 5.12	**< 0.001**
Treatment modalities and intent				
Surgery	1882	0.99	0.79, 1.25	0.944
Radiotherapy	1839	1.02	0.83, 1.25	0.839
Systemic therapy	1846	0.98	0.79, 1.22	0.884
Palliative treatment intent	1699	0.71	0.43, 1.19	0.186

We considered covariates with *P* < 0.1 in the univariate regression as candidates for multivariate logistic regression. Employing a bidirectional stepwise approach using the AIC for covariate selection resulted in a final model with a lower AIC (1,303.06) than the full model (1,315.40), indicating improved fit and parsimony. Multicollinearity was low across all models, with GVIF values in the final model ranging from 1.066 to 1.267.

Frailty, self-efficacy, social support, being well-informed about the illness and therapy options, the notion of being an active part in decision-making, and the perceived concordance of involved physicians’ actions were significant predictors in the multivariate regression models. Being informed about the illness and therapy options was confirmed to be the strongest associated with higher decision-making satisfaction (aOR: 5.73, 95% CI: 3.89 to 8.52), as was frailty for predicting lower decision-making satisfaction (aOR: 0.66, 95% CI: 0.48 to 0.90). The multivariate logistic regression models are displayed in Table 4.

**Table 4:** Multivariate logistic regression analysis.

	Full model including all predictors with *P* < 0.1 in univariate regression *N* = 1,478			Model after bi-directional stepwise selection process *N* = 1,478		
	aOR	95% CI	*P*	aOR	95% CI	*P*
Patient characteristics and ailments						
UICC staging*						
Stage I	–	–				
Stage II	1.24	0.88, 1.75	0.223			
Stage III	1.02	0.67, 1.58	0.929			
Stage IV	0.96	0.51, 1.86	0.904			
Need of care*	0.94	0.52, 1.73	0.841			
Depression*	1.10	0.77, 1.59	0.588			
Medication*	0.89	0.65, 1.22	0.452			
Frailty (TFI)	0.68	0.49, 0.94	**0.019**	0.66	0.48, 0.90	**0.008**
Patient autonomy and support						
Independently mobile*	1.44	0.75, 2.74	0.265			
Self-efficacy (ASKU scale)	1.62	1.29, 2.05	**< 0.001**	1.63	1.31, 2.04	**< 0.001**
Social support (FSozU scale)	1.32	1.05, 1.65	**0.015**	1.36	1.10, 1.67	**0.004**
Confidant available*	1.34	0.84, 2.13	0.214			
Knowledge and communication-related factors						
Knowledge of guidelines*						
Not heard about it	–	–				
Heard about it	0.94	0.65, 1.37	0.760			
Read it	0.80	0.56, 1.13	0.202			
Informed about illness and therapy	5.77	3.89, 8.63	**< 0.001**	5.73	3.89, 8.52	**< 0.001**
Inclusion in decision-making	2.93	2.15, 3.99	**< 0.001**	2.93	2.15, 3.98	**< 0.001**
Concordance of physicians’ actions	2.07	1.52, 2.79	**< 0.001**	2.01	1.49, 2.71	**< 0.001**
*Model fit*	*Nagelkerke’s R²: 0.382*,* AIC: 1315.399*			*Nagelkerke’s R²: 0.376*,* AIC: 1303.064*		

*Covariate initially considered but eliminated in bidirectional stepwise-selection process.

In a sex-stratified analysis, effect estimates for male and female participants agreed on the relevant predictors. Social support was not a significant predictor in the male-only model (aOR: 1.31, 95% CI: 0.86 to 2.00), but the effect estimate was similar to that in females (aOR: 1.35, CI: 1.10 to 1.66). Results of the sex-stratified analysis are presented in Supplementary Table S1. As a sensitivity analysis, we also fit a model containing all candidate predictors without prior selection (Supplementary Table S2); effect estimates were consistent with the main analysis in direction and magnitude, although frailty narrowly missed statistical significance in the full model (aOR: 0.72, 95% CI: 0.51 to 1.02).

## Discussion

In our sample of 2,011 patients aged 50 years and older with breast cancer or colorectal cancer, the vast majority (72.1%) were highly satisfied with the way in which therapy decisions are made. Predominantly high satisfaction scores align with other research on this topic. A cross-sectional study on prediagnosis care and care provided during treatment found mostly “excellent” marks in general patient satisfaction^34^. However, in that study, older age and male sex were significant contributors to high satisfaction, whereas we found no such association. In our sample, satisfaction did not differ by age, sex, cancer site, or cancer staging in multivariate analysis.

We identified factors from the domains of comorbidities, patient autonomy, social support, and knowledge and communication that were associated with patients’ perception of the decision-making process. Among the covariates corresponding to physical factors and comorbidities, only frailty was a significant predictor of lower satisfaction in multivariate analyses. Several mechanisms could plausibly account for this association. Frail patients may face a higher cognitive load during treatment consultations, considering not only the cancer-directed therapy itself but also its interactions with multimorbidity and competing priorities such as maintaining functional independence. Such conditions may increase the risk of feeling overwhelmed during the decision-making process. Standard models of shared decision-making have been shown to be insufficient for frail older patients, who benefit instead from goal-oriented counseling that explicitly involves informal caregivers and accommodates their personal priorities^35^. Such tailored approaches – together with the additional consultation time they require – can mitigate these challenges, but time pressure and the lack of geriatric-oriented communication skills in routine oncology practice are well-documented organizational barriers to their implementation^36^. It has been shown that patient fatigue is a predictor of poor overall patient satisfaction^37,38^. We did not specifically assess this factor because there is an overlap between fatigue and frailty. Fatigue often coincides with frailty^39^ and is part of the TFI and other frailty assessments^40^. Finally, we cannot rule out that frailty in our data partly acted as a proxy for other unmeasured factors such as health literacy, cognitive function, or functional decline, which are themselves known barriers to active participation in shared decision-making^36^.

On the other hand, the fact that higher UICC stage, need of care, and depression were not associated with lower satisfaction may indicate that medical professionals evidently already set up consultation and decision-making situations in a way that the potential unique needs of these patients are met. Seemingly unfavorable initial conditions do not predetermine a therapy decision situation perceived as unsatisfactory; nor, as our findings suggest, do treatment modality or therapeutic intent.

An analysis of breast cancer patients aged 70 and older showed that higher satisfaction with the decision-making process was associated with adherence to therapy guideline recommendations^25^. One explanation for this is that physicians may be more likely to provide strong and convincing recommendations in clear cancer cases that follow guideline-adherent therapy. Patients for whom a deviation from guideline recommendations is warranted may lack compelling, straightforward management recommendations, with the physician’s assurance that the treatment best suits their needs.

The findings from this study support the notion that disease severity alone does not necessarily lead to reduced satisfaction with medical communication and decision-making processes. Instead, the way consultations are conducted seems to play a significant role in shaping satisfaction outcomes. Physicians’ behaviors can influence the three most critical factors that contribute to high satisfaction with decision-making: perceived alignment and consistency of actions among multiple physicians, a sense of being actively and meaningfully included in the decision-making process, and a feeling of being thoroughly informed about the illness and available treatment options. Cancer patients predominantly prefer strong inclusion in treatment decisions, but preferences and perceived involvement do not always match^41^. This suggests that shared decision-making has not yet become a fully realized practice across all settings.

## Strengths and limitations

Our sample allowed us to examine factors contributing to satisfaction with the decision-making situation across gender, cancer site, and age (for people aged 50 and over), and to identify essential aspects of satisfactory treatment decision-making, highlighting the importance of medical communication in cancer treatment. However, the generalizability of our findings may be limited in several respects. Our sample skewed female, younger, and toward earlier disease stages. Additionally, patients more satisfied with their therapy decision-making process may have been disproportionately motivated to participate, potentially leading to an overestimation of satisfaction levels. Detailed non-responder analyses were not possible because additional clinical and personal data could only be obtained after enrolment.

Given the comprehensive nature of the overarching research project, the treatment decision situation was just one of several aspects we surveyed using an extensive questionnaire. This likely contributed to gaps in the responses, which accumulated in multivariate analyses. Missingness in UICC staging reflected incomplete or belated documentation in cancer registries. Registry patients with missing UICC staging did not differ from those with complete staging information with respect to age, sex, cancer site, or satisfaction rates.

The dichotomization of the seven-point satisfaction scale into a binary outcome entails a loss of information inherent to any such approach. The cut-off of 1–5 vs. 6–7 was chosen to reflect the skewed distribution of responses, with the majority of patients reporting high satisfaction. Sensitivity analyses supported the stability of the main findings across alternative specifications.

A further limitation concerns the cross-sectional nature of our satisfaction assessment. Because predictors such as perceived informedness, self-efficacy, and social support were measured concurrently with the satisfaction outcome, a causal relationship cannot be firmly established: patients who are more satisfied with the decision-making process may retrospectively rate their information level, sense of agency, or support more favorably. The associations reported here should therefore be interpreted with appropriate caution regarding causality.

## Conclusion

Physicians may enhance patient satisfaction by focusing on key factors such as keeping patients well-informed, actively involving them in decisions about their care, and coordinating effectively with other healthcare providers to foster a collaborative care environment. To further improve outcomes, tailored support systems should be implemented to meet the unique needs of patients, particularly those who are frail, have low confidence in managing their health, or lack adequate social support networks.

Further research should be conducted using both qualitative and quantitative methods that more closely examine the dynamics of decision-making and specifically account for potential sources of bias. In particular, longitudinal designs that assess predictors and satisfaction at separate time points would help establish temporal ordering and reduce the risk of reverse causation inherent to cross-sectional assessments. This will further clarify the conditions necessary for making decisions that address both the illness and the patient’s needs. Promising avenues include medical education, digital communication tools, and structured patient involvement frameworks. Special attention should be paid to situations in which the decision-making process was complicated and posed additional challenges for patients and physicians, e.g., when palliative treatment intent becomes a realistic prospect.

## Supplementary Information

Below is the link to the electronic supplementary material.

Supplementary Material 1

## Data Availability

The data that support the findings of this study are available from the corresponding author upon reasonable request.
