# Supplementary material for: Communication-related aspects drive senior patients’ satisfaction with the process of decision-making in cancer therapy
Source: Sci Rep. 2026 Apr 30;16:13917. doi: 10.1038/s41598-026-51157-y (PMC13133227; doi:10.1038/s41598-026-51157-y)
Supplement: Supplementary file 1 — Supplementary Material 1 [file 41598_2026_51157_MOESM1_ESM.docx]

Supplementary Table S1: Sex-stratified sensitivity analysis of the six predictors remaining after backward stepwise selection (multivariable logistic regression)

|  | **Male; N=349** | | | **Female; N= 1262** | | |
| --- | --- | --- | --- | --- | --- | --- |
|  | **aOR** | **95% CI** | **P** | **aOR** | **95% CI** | **P** |
| **Frailty** | 0.41 | 0.20, 0.84 | **0.014** | 0.72 | 0.53, 0.99 | **0.044** |
| **Self-efficacy expectations** | 1.76 | 1.05, 3.00 | **0.033** | 1.52 | 1.23, 1.90 | **<0.001** |
| **Social support** | 1.31 | 0.86, 2.00 | 0.208 | 1.35 | 1.10, 1.66 | **0.005** |
| **Information about illness and therapy** | 3.70 | 1.71, 8.10 | **<0.001** | 5.80 | 3.89, 8.76 | **<0.001** |
| **Inclusion in decision-making** | 2.36 | 1.17, 4.74 | **0.016** | 3.03 | 2.22, 4.13 | **<0.001** |
| **Concordance of physicians’ actions** | 2.46 | 1.27, 4.72 | **0.007** | 1.88 | 1.39, 2.53 | **<0.001** |
|  | | | | | | |

Supplementary Table S2: Full model without prior variable exclusion

|  | Full model including all predictors, no prior exclusion; N = 1,309 | | |
| --- | --- | --- | --- |
|  | **aOR** | **95% CI** | **P** |
| *Patient characteristics and ailments* | | | |
| Age | 1.00 | 0.98, 1.02 | 0.900 |
| Sex |  |  |  |
| Female | — | — |  |
| Male | 1.16 | 0.67, 2.00 | 0.587 |
| Cancer site |  |  |  |
| Breast cancer | — | — |  |
| Colorectal cancer | 1.35 | 0.81, 2.27 | 0.257 |
| UICC staging |  |  |  |
| Stage I | — | — |  |
| Stage II | 1.21 | 0.84, 1.76 | 0.303 |
| Stage III | 0.86 | 0.53, 1.41 | 0.544 |
| Stage IV | 1.05 | 0.44, 2.56 | 0.921 |
| Need of care | 1.04 | 0.54, 2.04 | 0.909 |
| Depression | 1.03 | 0.70, 1.53 | 0.895 |
| Medication | 0.81 | 0.57, 1.15 | 0.228 |
| Frailty (TFI) | 0.72 | 0.51, 1.02 | 0.062 |
| *Patient autonomy and support* | | | |
| Independently mobile | 1.20 | 0.58, 2.47 | 0.611 |
| Self-efficacy (ASKU scale) | 1.58 | 1.23, 2.03 | **<0.001** |
| Social support (FSozU scale) | 1.36 | 1.07, 1.72 | **0.013** |
| Confidant available | 1.25 | 0.75, 2.05 | 0.392 |
| *Knowledge and communication-related factors* | | | |
| Knowledge of guidelines |  |  |  |
| Not heard about it | — | — |  |
| Heard about it | 0.88 | 0.59, 1.31 | 0.517 |
| Read it | 0.75 | 0.51, 1.09 | 0.129 |
| Informed about illness and therapy | 5.96 | 3.89, 9.24 | **<0.001** |
| Inclusion in decision-making | 3.15 | 2.25, 4.40 | **<0.001** |
| Concordance of physicians’ actions | 2.10 | 1.51, 2.91 | **<0.001** |
| *Treatment modalities and intent* | | | |
| Surgery | 1.03 | 0.70, 1.51 | 0.865 |
| Radiotherapy | 1.37 | 0.97, 1.95 | 0.078 |
| Systemic therapy | 1.16 | 0.83, 1.63 | 0.382 |
| Palliative treatment intent | 0.63 | 0.26, 1.61 | 0.320 |
